# The introduction of a regional Norwegian HEMS coordinator: an assessment of the effects on response times, geographical service areas and severity scores

**DOI:** 10.1186/s12913-022-08337-z

**Published:** 2022-08-10

**Authors:** Ole Erik Ulvin, Eivinn Årdal Skjærseth, Helge Haugland, Kjetil Thorsen, Trond Nordseth, Marie Falch Orre, Lars Vesterhus, Andreas Jørstad Krüger

**Affiliations:** 1 Department of Research, Norwegian Air Ambulance Foundation, Oslo, Norway; 2 Department of Circulation and Medical Imaging, Faculty of Medicine and Health Sciences, Norwegian University of Science and Technology (NTNU), Trondheim, Norway; 3 Department of Emergency Medicine and Pre-Hospital Services, St. Olav`s University Hospital, Trondheim, Norway; 4 Department of Anaesthesia and Intensive Care Medicine, St. Olav`s University Hospital, Trondheim, Norway; 5 Department of Research and Development, Division of Emergencies and Critical Care, Oslo University Hospital, Oslo, Norway; 6 Department of Civil and Environmental Engineering, Master’s Degree Programme of Engineering and ICT, Norwegian University of Science and Technology (NTNU), Trondheim, Norway

**Keywords:** HEMS coordination, Response time, Interrupted time series, Convex hull

## Abstract

**Background:**

Due to unwanted delays and suboptimal resource control of helicopter emergency medical services (HEMS), regional HEMS coordinators have recently been introduced in Norway. This may represent an unnecessary link in the alarm chain, which could cause delays in HEMS dispatch. Systematic evaluations of this intervention are lacking. We wanted to conduct this study to assess possible changes in HEMS response times, mission distribution patterns and patient characteristics within our region following this intervention.

**Methods:**

We retrospectively collected timeline parameters, patient characteristics and GPS positions from HEMS missions executed by three regional HEMS bases in Mid-Norway during 2017–2018 (preintervention) and 2019 (postintervention). The mean regional response time in HEMS missions was assessed by an interrupted time series analysis (ITS). The geographical mission distribution between regional HEMS resources was assessed by a before-after study with a convex hull-based method.

**Results:**

There was no significant change in the level (-0.13 min/month, *p* = 0.88) or slope (-0.13 min/month, *p* = 0.30) of the mean regional response time trend line pre- and postintervention. For one HEMS base, the service area was increased, and the median mission distance was significantly longer. For the two other bases, the service areas were reduced. Both the mean NACA score (4.13 ± SD 0.027 vs 3.98 ± SD 0.04, *p* < 0.01) and the proportion of patients with severe illness or injury (NACA 4–7, 68.2% vs 61.5%, *p* < 0.001) were higher in the postintervention group.

**Conclusion:**

The introduction of a regional HEMS coordinator in Mid-Norway did not cause prolonged response times in acute HEMS missions during the first year after implementation. Higher NACA scores in the patients treated postintervention suggest better selection of HEMS use.

## Background

The dispersed population and long prehospital distances make helicopter emergency medical services (HEMS) crucial in northern Scandinavia [1]. However, operating HEMS around the clock also calls for economic and safety considerations [2, 3]. In 2018, helicopters and airplanes completed 18 600 air ambulance missions in Norway at a cost of nearly 100 million Euros [4]. Challenging operational contexts due to darkness and bad weather might add risk to both patients and crews [5]. Moreover, unnecessary dispatch of HEMS to the wrong patients might lead to concurrencies or exceeded duty time, thus affecting availability of the service [6]. When evaluating the use of this specialized service, medical patient benefit must therefore be weighed against financial costs and potential risks related to flight operations [7]. Hence, appropriate resource utilization of HEMS has become an increasingly important topic in prehospital emergency research [2, 7–11]. The way HEMS resources within a region are dispatched and coordinated is an essential part of this and a subject for current research [12–14]. Due to the complexity of several contextual factors, such as perceived severity, available alternative resources, weather conditions and mission location involved in the dispatch process, evidence for the benefit of specific HEMS dispatch criteria is scarce [14, 15]. As such, organizational factors of HEMS coordination in emergency medical communication centres (EMCCs), including the education of dispatchers and HEMS notification procedures, also become important to consider when assessing optimal HEMS utilization [16].

In 2014, the South-Eastern Norway Regional Health Authority, as the first of Norway’s four health authorities, established a regional HEMS EMCC [13, 17]. Suboptimal control over HEMS flights in the region and unwanted HEMS delays for EMCCs without HEMS coordination were the main reasons for this system change. During the terror attack on July 22^nd^ in Oslo in 2011, flight-following systems (a safety measure to ensure urgent response in case of emergencies) were also found to be inadequate [13, 17–19]. In addition to their basic education as nurses, paramedics or emergency medical technicians, selected EMCC operators were then specially trained on different aspects of HEMS operations (including emergency medicine, rescue techniques and crew resource management principles) to staff the regional HEMS EMCC [19]. In subsequent years, a HEMS EMCC was established in the remaining three regions, including the Central Norway Regional Health Authority, in January 2019 [20].

In this new system, the HEMS dispatch process starts with an emergency call responded to by a local EMCC. If needed, a local ambulance is alarmed. The local general practitioner (GP) is alarmed if indicated by the EMCC guidelines. Based on national guidelines for HEMS activation [21], local operators also decide if HEMS dispatch is indicated. If so, the regional HEMS coordinator is contacted and alarms the most appropriate HEMS unit. Similar to previous dispatch routines before the HEMS coordinator introduction, the final decision on whether to accept or reject a mission is made by the HEMS crew. This decision is based on both medical evaluations by the HEMS physician and operational considerations (like weather conditions and duty time limitations) by the HEMS pilot.

Activation time, i.e., the time from emergency call to HEMS take off, has been shown to be strongly influenced by the number of intermediators involved in emergency calls [22]. As such, adding an extra HEMS coordinator link could cause HEMS dispatch delay, which might affect patient outcome in time-critical settings. Until now, evaluations of this intervention have been limited to simply measuring the extra call delay, which was found to be 35 s on average during the first 6 months after implementation in one region [13]. Prior to the intervention, this potential delay was thought to be outweighed by improved coordination of regional HEMS resources, which could affect response times, mission distribution and alarming procedures [13, 17]. As with any major organizational change in critical parts of the emergency medical services (EMS) [23], this intervention should therefore be more thoroughly evaluated.

The main aim of this study was to evaluate possible changes in response time for patients treated by HEMS in mid-Norway after the introduction of a regionalized HEMS coordinator. The secondary aim of the study was to assess possible changes in service areas, in-flight scrambles and patient characteristics for each regional HEMS unit following this intervention.

## Methods

### Study setting—hospital organization and HEMS resources in Central Norway

The Central Norway Health authority is a state-owned company responsible for specialist healthcare services in the region of Central Norway. This includes eight hospitals, with a population of approximately 730 000 inhabitants distributed over an area of approximately 56 000 square kilometres [24].

The rotor wing air ambulance resources in the region consist of the HEMS bases in Trondheim and Ålesund and one search and rescue (SAR) helicopter at Ørland Air Force Base. The HEMS base in Trondheim operates an Airbus Helicopter H145 (start-up time 3 min, cruising speed 120 knots), while the Ålesund base operates an Augusta Westland 139 (start-up time 2 min, cruising speed 145 knots). A Westland Sea King helicopter (start-up time 10 min, cruising speed 110 knots) was until May, 2021 stationed at Ørland Air Force Base and operated by the 330 squadron of the Royal Norwegian Air Force. This helicopter performed both SAR and ambulance missions, and was in May 2021 replaced by the new Augusta Westland AW101 Sar Queen. Helicopters from other adjacent regions, such as Brønnøysund and Dombås, also regularly transport patients to the hospitals in the region.

In HEMS operations, an *acute mission* implies medical urgency with the need for an immediate emergency response. A *primary mission* is defined as a mission occurring out-of-hospital where “air ambulance and/or rescue helicopter attend the patient directly at the scene and perform transport from the scene to a health care facility” [25]. *In-flight scrambles* are alarms received when the helicopter is already airborne and were in this study defined as an abnormally short (< 2 min) interval between the HEMS alarm and HEMS take off time. This rapid response is not possible to acquire unless the helicopter is already airborne.

### The intervention

On January 7^th^, 2019, the new HEMS coordinator located at the regional EMCC at Trondheim University Hospital started coordinating all HEMS activity in the Central Norway Regional Health Authority.

### Primary and secondary outcomes

**Fig. 1:**
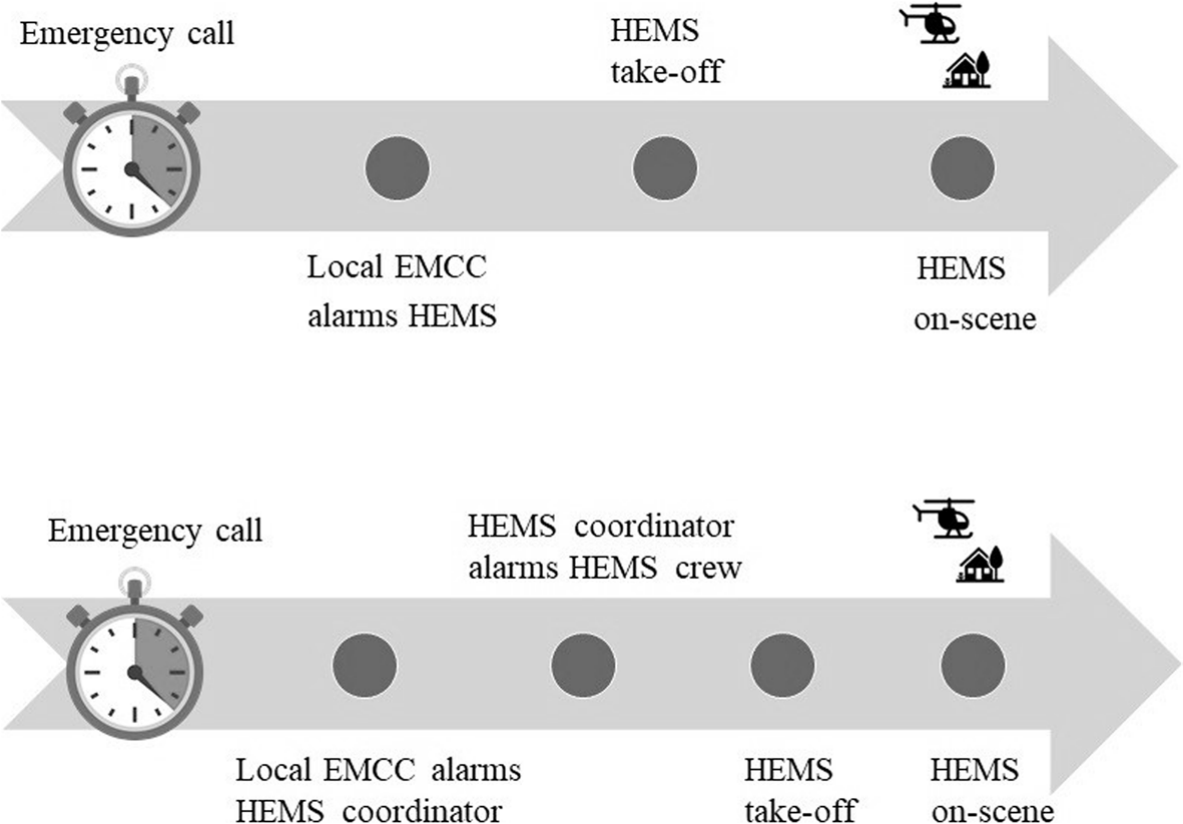
HEMS activation algorithm in the old (upper) and new (lower) systems

The primary outcome was the *mean regional response* time in acute primary HEMS missions in the Central Norway Regional Health Authority. *Response time* (Fig. 1) was defined as the time from the local EMCC operator alarming the HEMS crew to arrival of the HEMS crew on-scene (before 2019, preintervention), and the time from the HEMS coordinator was alarmed by the local EMCC to arrival of the HEMS crew on scene (from January 7^th^ 2019, postintervention). By this definition, the earliest parts of the timeline involving emergency calls, local EMCC and dispatch decisions were excluded. Potential changes in response time could therefore be addressed to the intervention.

The secondary outcomes were whether there was a change in the geographical mission distribution or the number of in-flight scrambles for the three regional HEMS bases in Mid-Norway following the intervention.

### Inclusion and exclusion criteria

All completed, primary, acute HEMS missions executed by the three helicopter bases (Trondheim, Ålesund, Ørland) in the Central Norway Regional Health Authority during 2017–2019 were included. Exclusion criteria included cancelled or aborted missions, missions performed by rapid response cars, search-and-rescue (SAR) missions, secondary missions (interhospital transfers) and missions without patient contact.

### Data sources, collection and cleaning

We collected data retrospectively from the EMCC database AMIS and the HEMS database LABAS. *AMIS* (CSAM Health AS, Oslo, Norway) is an emergency medicine information system used in every EMCC in Norway. It contains a variety of EMS data, including information about emergency calls, ambulance dispatch, patient status reports and timeline data. *LABAS* (Normann IT, Trondheim, Norway) is the dedicated operational database and medical record generator of the Norwegian HEMS service.

For each HEMS mission, we obtained the date of mission, patient characteristics (age, gender, diagnosis, NACA severity score [26]), timeline parameters (time of emergency call, EMCC operator decision of HEMS dispatch, HEMS coordinator contacted, HEMS alarm, HEMS take-off, HEMS on-scene) and GPS position of the HEMS mission. After data were collected, missions with missing data and/or extreme outliers were reviewed by the medical leaders of each HEMS base to correct obvious errors in the databases, if possible. If multiple patients were transported by the same helicopter on a mission, this was registered as a single HEMS mission. The extra patients were included in the descriptive analyses. In the event of different registered arrival times of patients in a mission including two or more patients, the timestamp for the first patient contact was utilized.

### Analyses

#### Primary outcome

To assess if there was a change in mean regional response time after the HEMS coordinator introduction, we performed an interrupted time series (ITS) analysis [27]. This method was chosen due to its ability to account for both natural trends and seasonal variations in response time, which was expected, and also distinguish between immediate (level change) and trend (slope change) effects following the intervention. The main hypothesis was a gradual slope decrease in mean response time after the intervention had been established.

Response times from each mission were aggregated in monthly intervals, creating a mean monthly regional HEMS response time. January 2019 was excluded from the analysis because the intervention was introduced this month. The mean regional response time for December 2019 represented an extreme outlier (3,4 standard deviations from the mean) and was therefore also excluded. Hence, 24 data points (months) were included before the intervention and 10 after. A mean value was found to be preferable to the median as the measure of central tendency due to the hypothesis that a regional HEMS coordinator would have the largest impact on medium- to long-range missions.

The response time was modelled as an ITS using a segmented linear regression model with a discontinuity at the intervention point and a seasonal ARIMA process [28] (SARIMA) as the error term. The optimal model for the mean regional response time *f* as a function of time *t* was selected based on fit and parsimony (using R^2^ and AICc) and expressed as

$$f\left(t\right)={\beta }_{0}+{\beta }_{1}t+{\beta }_{2}u\left(t-{t}_{pi}\right)+{\beta }_{3}\left(t-{t}_{pi}\right)u\left(t-{t}_{pi}\right)+{{\beta }_{4}I}_{\text{winter}}\left(t\right)+{\varepsilon }_{MA\left(1\right)}\left(t\right)$$

where *β*_*0*_ represents the response time at t = 0 (January 2017), *β*_*1*_ is the trendline coefficient of the preintervention period, *β*_*2*_ is the level change in response time immediately postintervention, *β*_*3*_ is the trend change between the post- and preintervention periods, and *β*_*4*_ represents the effect of seasonality. ${I}_{\text{winter}}\left(t\right)$ is the indicator function with a value of 1 from October to March and 0 otherwise, and ${\varepsilon }_{MA(1)}$ is the stochastic error term modelled as a first-order moving average (MA) process. To determine if there was a significant change in level and/or trend following the intervention, we performed hypothesis tests on *β*_*2*_ and *β*_*3*_.

#### Secondary outcomes

The geographical service areas of regional HEMS bases were analysed by a convex hull-based method, which recently has been proposed to serve as a standard method for defining HEMS operating areas [29]. A convex hull is defined as *“the smallest geometric shape which contains a predetermined set of points”* [29]. GPS locations of each mission from the AMIS database were plotted in commercially available map software (ArcGIS® Pro version 2.6, Esri GIS Mapping Software Inc., Redlands, California, USA). Based on the locations of the three regional HEMS bases, geodesic distances (i.e., the shortest path between two points on a curved surface) for the missions were calculated. The 5% of missions with the longest distance were defined as outliers and excluded from further analyses. The remaining missions were then used to create a convex hull for each HEMS base. The analyses were performed for each base before and after the introduction of the regional HEMS coordinator, and intergroup comparisons were made regarding the median mission distance and size of each unit’s actual service areas.

### Epidemiological data and statistical software

The differences in the patient populations before and after the intervention were analysed by Mann–Whitney U test, Student`s t-test or Pearson`s chi-square test, as appropriate, with a chosen significance level of 0.05. Data are reported as the mean with standard deviation (SD), median with interquartile range (IQR) or proportions, as appropriate.

Data were stored on a secure server at Central Norway Regional Health Authority`s IT department (HEMIT). Statistical analyses were performed using IBM Statistics SPSS 27 (IBM Corp. Released 2020. IBM SPSS Statistics for Windows, Version 27.0. Armonk, NY: IBM Corp), R Statistics 4.0.4 (R Core Team 2013, R Foundation for Statistical Computing, Vienna, Austria) and Microsoft Excel (Microsoft Office 365 ProPlus, Microsoft Corporation, USA).

## Results

**Fig. 2:**
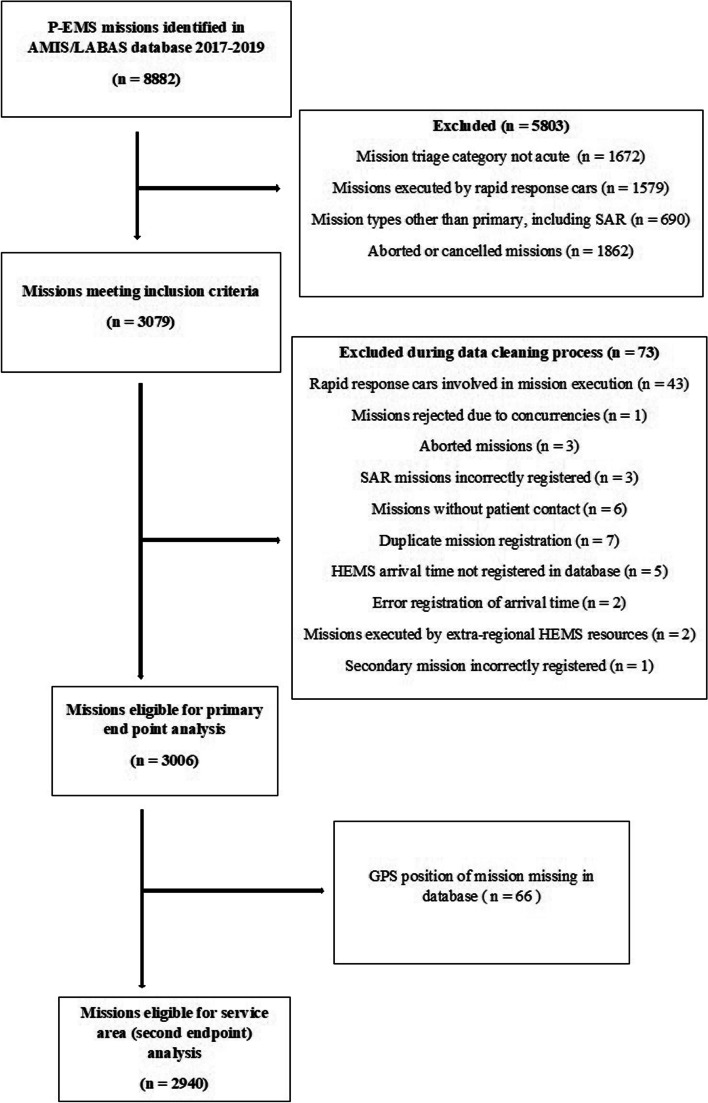
Flow chart of inclusion and exclusion criteria

A total of 3006 missions involving 3059 patients were eligible for response time analysis, and 2940 missions were eventually included in the service area map analyses (Fig. 2).

### Patient demographics

Sixty-four and 66% of patients were male, with median ages of 55 (IQR 42) and 59 (IQR 37) years in the pre- and postintervention groups, respectively (*p* = 0.01) (Table 1). The mean NACA score was higher in the postintervention group (4.13 ± SD 0.027 vs 3.98 ± SD 0.04, *p* < 0.01). The proportion of patients with severe illness or injury (defined as a NACA score of 4 or higher) was higher in the postintervention group (68.2% vs 61.5%, *p* < 0.001). Patient diagnoses in both groups were dominated by cardiovascular diseases and traumas*.*

**Table 1:** Patient characteristics in HEMS missions pre and post HEMS coordinator introduction

	**Preintervention**				**Postintervention**				**Diff. in mean/**	**Missing**	***P*-value**
	n	%			n	%			**median/%**	n	
**Total *n* = 3059**	**2139**	**69.9**	Mean (± SD)	Median (IQR)	**920**	**30.1**	Mean (﻿±SD)	Median (IQR)			
**Age (years)**											
All patients	2133	99.7		55.0 (42)	918	99.8		59.0 (37)	4.0	8	0.011^3^
**Gender**										1	0.35^5^
Male	1358	63.5			607	66.0					
Female	780	36.5			313	34.0					
**NACA-score**^**1**^										1	
All patients	2139	100	3.98 (± 0.027)	4.0 (2)	919	99.9	4.13 (± 0.04)	4.0 (2)	0.15/-		0.003^4^
NACA 4–7	1316	61.5			627	68.2			6.7%	1	< 0.001^5^
**ICD-10 Codes**^**2**^	**2138**	**99.9**			**919**	**99.9**				2	
G00-G99	85	4.0			29	3.2					
I00-I99	758	35.4			365	39.7					
J00-J99	121	5.7			57	6.2					
M00-M99	33	1.5			5	0.5					
O00-O9A	49	2.3			15	1.6					
R00-R99	319	14.9			115	12.5					
S00-T88	631	29.5			279	30.3					
Other diagnoses	142	6.6			54	5.9					

^1^National Advisory Committee for Aeronautics. NACA 0 No injury or illness; NACA 1 Injuries/diseases without any need for acute physician care; NACA 2 Injuries/diseases requiring examination and therapy by a physician, but hospital admission is not indicated; NACA 3 Injuries/disease without acute threat to life but requiring hospital admission; NACA 4 Injuries/diseases that can possibly lead to deterioration of vital signs; NACA 5 Injuries/diseases with acute threat to life; NACA 6 Injuries/diseases transported after successful resuscitation of vital signs; NACA 7 Lethal injuries or diseases (with or without resuscitation attempts)

^2^G00-G99:Diseases of the nervous system; I00-I99: Diseases of the circulatory system; J00-J99: Diseases of the respiratory system;M00-M99:Diseases of the musculoskeletal system and connective tissue; O00-O9A: Pregnancy, childbirth and the puerperium; R00-R99: Symptoms, signs and abnormal clinical and laboratory findings, not elsewhere classified; S00-T88: Injury, poisoning and certain other consequences of external causes

^3^ Mann–Whitney U-test

^4^ Student’s t-test

^5^ Chi-square Test

### Primary outcome

**Fig. 3:**
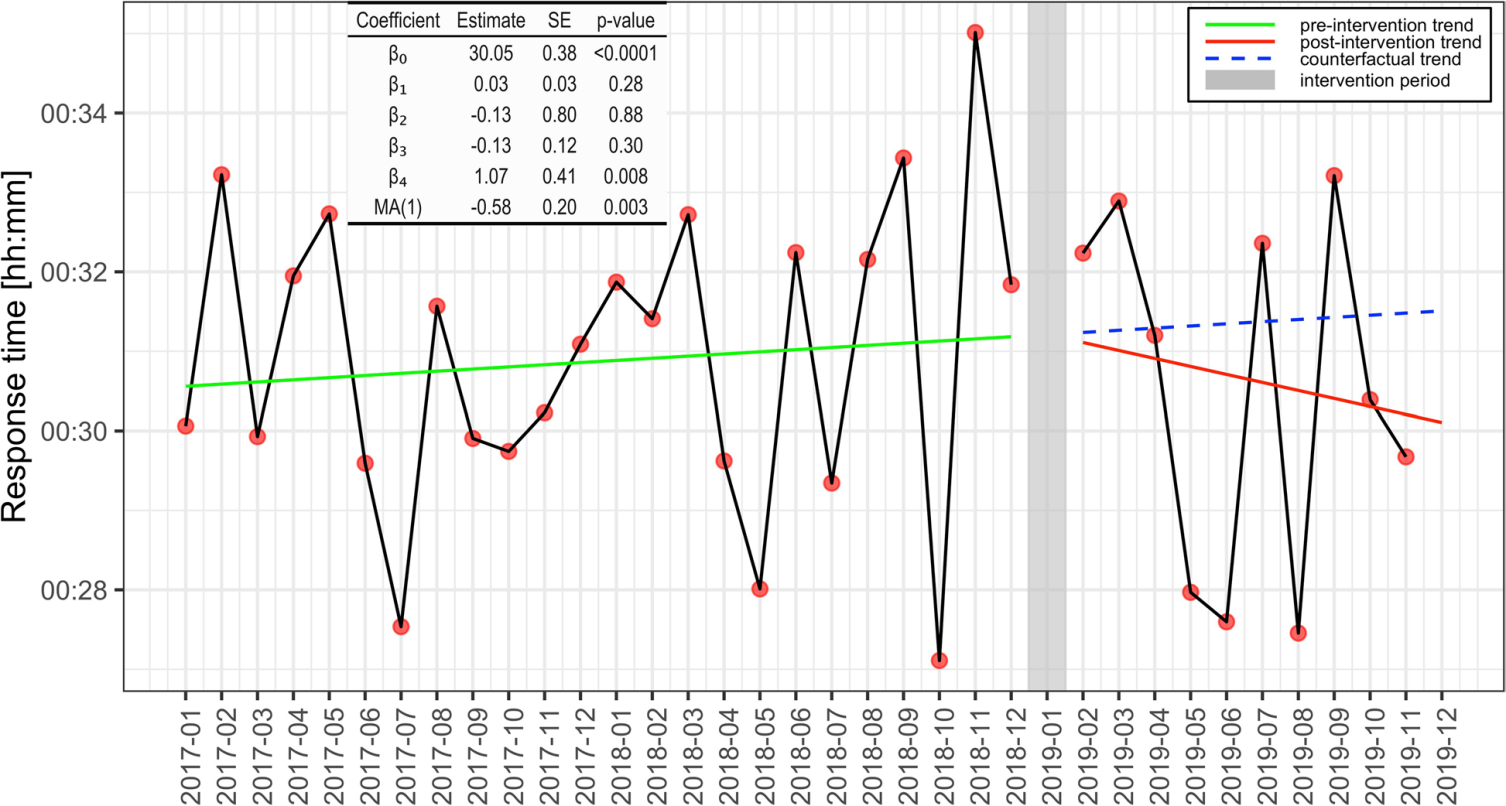
Interrupted time series analysis plot of regional response time before and after HEMS coordinator introduction. β_1_ is the trendline coefficient of the preintervention period, β_2_ is the level change in response time immediately postintervention, β_3_ is the trend change between the post- and preintervention periods, and β_4_ represents the effect of seasonality. The counterfactual trend line (blue) represents the expected response time development in case the intervention was not implemented. The difference in slope and level of the red and blue trend lines represent the effect of the intervention

In 88.2% (pre) and 86.7% (post) of the missions in the region, the responding HEMS unit reached the patient within 45 min (Table 2). The ITS analysis found a significant seasonal effect with a 1.1-min increase in the mean response time (*p* < 0.01) during the winter months (October through March). A visual trend towards decreasing response time was seen postintervention (Fig. 3), but there was no significant change in either the level (-0.13 min/month, *p* = 0.88) or slope (-0.13 min/month, *p* = 0.30) of the trend line. In summary, the mean regional response time trend did not change significantly after the introduction of the HEMS coordinator.

**Table 2:** Mission characteristics pre and post HEMS coordinator introduction

**Total *N* = 3006**		**Preintervention**	**Postintervention**	**Difference pre/post**		***P*-value**
	Unit			Unit		
**Mission distribution within region**	% (n)			%		0.07 ^1^
Trondheim HEMS		52.8 (1110)	49.2 (445)		-3.6	
Ålesund HEMS		31.7 (667)	36.0(326)		4.3	
Ørland SAR		15.4 (324)	14.8 (134)		-0.6	
**Mean response time**	mm:ss (± SD)					
All bases		31:03 (± 24 s)	31:56 (± 33 s)			
Trondheim HEMS		32:14 (± 30 s)	32:53 (± 48 s)			
Ålesund HEMS		26:31 (± 33 s)	29:21 (± 45 s)			
Ørland SAR		36:22 (± 98 s)	35:05 (± 105 s)			
**Missions within 45 min threshold**	% (n)			%		
All bases		88.2 (1853)	86.7 (785)		-1.5	0.26 ^1^
Trondheim HEMS		87.7 (973)	87.4 (389)		-0.3	0.90 ^1^
Ålesund HEMS		93.4 (623)	88.7 (289)		-4.7	0.01 ^1^
Ørland SAR		79.3 (257)	79.9 (107)		0.6	0.90 ^1^
**In-flight scrambles**	% (n)			%		
All bases		12.6 (265)	11.2 (101)		-1.4	0.26 ^1^
Trondheim HEMS		6.9 (77)	5.2 (23)		-1.7	0.20 ^1^
Ålesund HEMS		19.2 (128)	16.6 (54)		-2.6	0.32 ^1^
Ørland SAR		18.5 (60)	17.9 (24)		-0.6	0.90 ^1^
**Median geodesic mission distance**	km/IQR (n)			%/km(95% CI^3^)		
All bases		56/54.4 (2044)	59.1/55.9 (896)		5.5/3.1 (-1.3, 4.5)	0.29 ^2^
Trondheim HEMS		66.8/49.5 (1080)	72.6/45.6 (441)		8.7/5.8 (2.5,10.1)	< 0.001^2^
Ålesund HEMS		51/46.3 (651)	46.9/51.9 (322)		-8.0/-4.1 (-8.7,0.2)	0.068 ^2^
Ørland SAR		41.4/40.9(313)	42.3/45.5 (133)		2.0/0.9 (-5.2,7.4)	0.75 ^2^
**Convex hull (95% percentile) area**	km^2^			%/km^2^		
Trondheim HEMS		41553	43198		4.0/1645	
Ålesund HEMS		49755	28785		-42.1/-20970	
Ørland SAR		32946	23739		-27.9/-9207	

^1^ Chi-Square Test

^2^ Mann–Whitney U-test

^3^ 95% CI for difference in medians calculated by the Hodges-Lehmann estimation

## Secondary outcomes

**Fig. 4:**
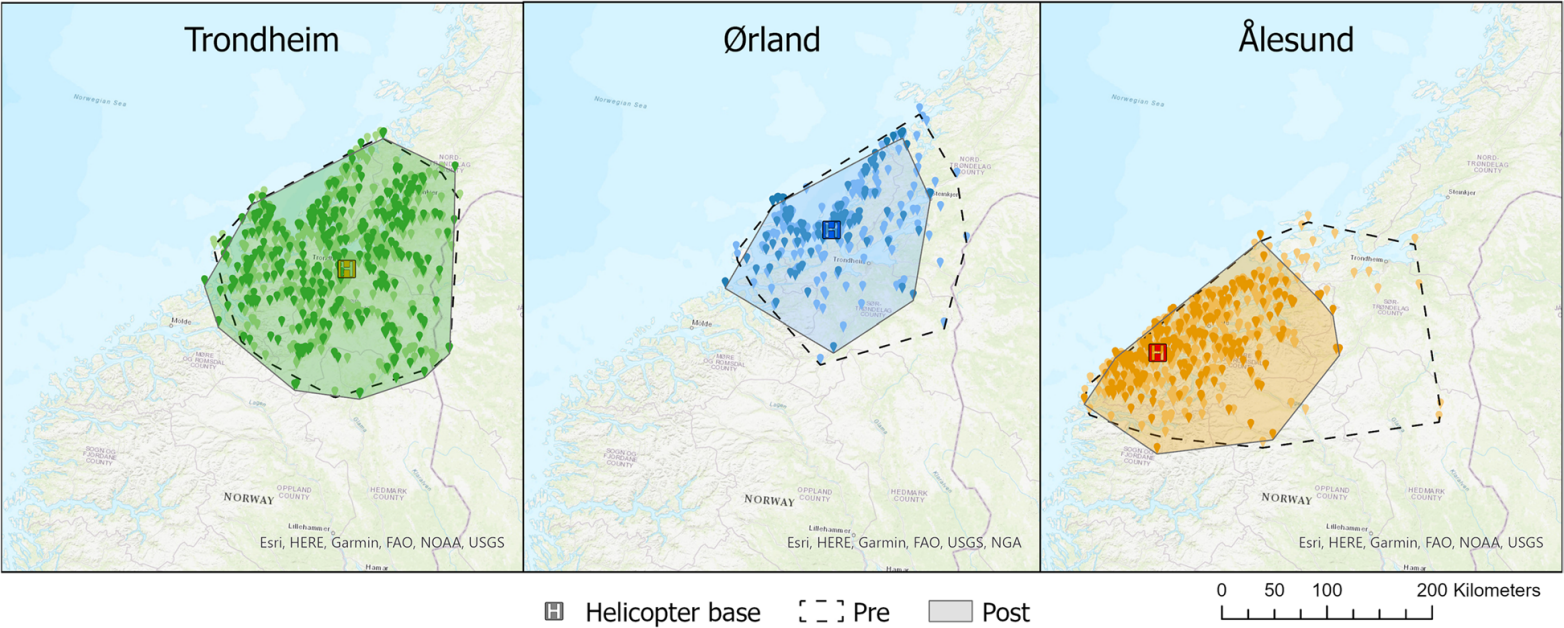
Convex hull and mission location plots for regional helicopters in the Central Norway Regional Health Authority before and after HEMS coordinator introduction

The service area for the Trondheim HEMS base was increased after the intervention (+ 4.0%) and decreased for the other regional bases (-42.1% for Ålesund HEMS and -27.9% for Ørland SAR) (Fig. 4). As presented in Table 2, the median geodesic mission distance for Trondheim HEMS was significantly higher postintervention (median distance 72.6 km vs 66.8 km, *p* < 0.001). There was no significant difference pre- and postintervention regarding the median mission distance for all bases summarized. The proportion of in-flight scrambles did not differ significantly between the pre- and postintervention groups (12.6% vs 11.2%, *p* = 0.26).

## Discussion

In this pre-post study, we found no significant trend change in the mean regional response time in acute, primary HEMS missions after the HEMS coordinator introduction. There was no change in the proportion of in-flight scrambles before and after the intervention for the regional helicopters. A significantly higher mean NACA score and a higher proportion of patients with severe illness or injury (NACA 4–7) were found in the postintervention group. For two of the bases (Ålesund and Ørland), we found a smaller geographical service area after the intervention but no significant change in median mission distance. For the Trondheim helicopter base, the service area was larger postintervention, and the median mission distance was significantly longer.

### Response time and ITS analysis

Shortening response time, yet not compromising flight safety, has been emphasized by several operators in European HEMS [22]. Although evidence for a clear relationship between response times and patient outcome has been scarce, time variables should be a part of a multidimensional quality measurement of prehospital care [30–32]. According to national guidelines regarding response times for HEMS in Norway, the goal is to reach 90% of the population within 45 min of alarm time [33]. In our study, the proportions of missions meeting this goal were 88.2% and 86.7% pre- and postintervention, respectively. Due to varying population density throughout the region, data based on completed missions only do not necessarily reflect the actual response time for the entire regional population. However, these numbers might indicate proper HEMS population coverage in Central Norway.

The mean response times in our analyses were comparable to previous Norwegian studies of the topic [34, 35]. For Ålesund HEMS, the number of missions within the 45 min threshold were significantly reduced postintervention, and the mean response time was increased. This could indicate changes for Ålesund HEMS not captured by the regional response time ITS analysis. However, although calculating mean response time is valuable for descriptive purposes, this method does not consider important features of interrupted time series analysis like historical trends, seasonality and autocorrelation [27]. These findings must therefore be interpreted with caution, but could indicate the need for a subgroup ITS analysis for each HEMS base in the region. This was, however, considered to be beyond the scope of this study. The overall longer mean response time and lower proportion of patients reached within 45 min for Ørland SAR base is primarily assumed to be due to longer activation time for the SAR helicopter compared to Trondheim and Ålesund HEMS.

Interrupted time series (ITS) is considered to be among the strongest quasi-experimental research designs for evaluating health care interventions when randomization is not possible [36]. The introduction of the HEMS coordinator at a clearly defined time point, an expected rapid change in response time after the implementation, easily accessible data points before and after the intervention and relatively stable historical trends of response time made this analysis preferable for this study. Although ITS analysis is considered robust against common threats of validity, potential cointerventions implemented simultaneously and related to the outcome should be evaluated [36, 37]. Parallel to the HEMS coordinator introduction in January 2019, the HEMS dispatch criteria of the region were clarified. For trauma patients, a hit in the anatomical or physiological criteria of the national trauma programme was now considered necessary for HEMS activation. In haemodynamically stable patients with acute myocardial infarction, HEMS was not dispatched until ECG confirmed an ST-elevation infarction, and there was an expected significant time gain to the nearest hospital with percutaneous coronary intervention (PCI). We assume that this clarification of dispatch criteria has affected regional HEMS activity, although no formal evaluation of this has been carried out yet. However, we consider the new criteria to be mainly influencing the dispatch decision – does this patient require HEMS? Identical to the situation before 2019, this decision fully relies on the local EMCC operator (Fig. 1). Thus, the regional HEMS coordinator should not be directly affected by the new dispatch criteria. We therefore consider this cointervention to be of minor importance when evaluating HEMS response times in the region.

On July 1^st^, 2018, the operator of one of the three HEMS bases included in the study (Ålesund base) was changed from Lufttransport AS to Norsk Luftambulanse AS due to a national tender competition in Norwegian HEMS. Following this, a new helicopter (Airbus Helicopters H145) was established as the spare helicopter, intended to be used when the main helicopter is out of service due to planned maintenance. The main helicopter at the Ålesund base was still Augusta Westland 139, as it was before the change of operator. Due to small differences between the helicopters regarding speed and start-up times, we consider this to have minor impact on our analyses.

The ITS analysis showed no evidence of altered mean response time after adding an extra link in the EMCC chain. The visual decreasing trend (Fig. 3) postintervention is promising, but the conclusions are limited by a relatively short postintervention observation interval. Hence, a follow-up analysis with a longer postintervention interval would seem reasonable to perform. Most importantly, at this early postintervention stage, there was no significant increase in mean regional response time following the intervention.

### Service areas and in-flight scrambles

The geographical distance to the patient is the single most important factor in the determination of response time in a HEMS mission. Hence, a potential change in response times following the new HEMS coordinator could be associated with modified geographical dispersion of missions between the regional helicopters. For Trondheim HEMS, we found a longer median mission distance and a larger service area postintervention. Additionally, the service areas for Ålesund HEMS and Ørland SAR were reduced. The unbalanced study periods with notably fewer missions in the dataset postintervention, and the exclusion of primary missions performed in adjacent health regions, challenge draw robust conclusions from these changes. Also, a minor change in long-range missions might lead to a relatively large reduction in the service area when using the convex-hull method. However, these findings might indicate that Trondheim HEMS is more frequently used in the overlapping areas between bases. Possible explanations for this might be the general trend of centralization in medical care towards tertiary hospitals [38] (St Olav`s University Hospital is located in Trondheim) and the geographical and organizational proximity of the HEMS coordinator to the Trondheim HEMS base.

Another potential effect of a regional HEMS coordinator could be an increased number of in-flight scrambles due to improved coordination and real-time overview of the resources within a region. We found no significant difference in the frequency of these alarms pre- and postintervention. The detection of in-flight scrambles based only on a short activation time interval (2 min or less) might not be sufficiently sensitive, and other methods to register these missions should therefore be established.

### Epidemiological data of patients in HEMS missions

A significantly higher mean NACA score and proportion of patients with severe illness or injury were found in the postintervention group. However, the large sample size of the dataset can cause low p-values for clinically less relevant outcomes and should be interpreted with caution [39] and assessed together with estimated effect sizes. The mean NACA score in the population is comparable with previous findings [40]. The regional HEMS coordinator was not involved in the HEMS dispatch decision, and changes in the NACA distribution amongst HEMS patients are therefore presumably due to the dispatch criteria revision and not the HEMS coordinator introduction itself. The numbers do, however, indicate a positive trend in the ability of the prehospital chain to identify patients with medical benefit from HEMS services and to facilitate the use of HEMS in these patient groups.

### Strengths

The systematic analysis of a major health system intervention based on a complete dataset and a robust method in a nonrandomizable setting are the major strengths of the study. We argue that ITS might prove useful for any intervention separated in time and that this way of describing service areas might be relevant for many topics in prehospital emergency medicine.

### Limitations

Using monthly aggregated data to calculate a mean regional response time does not account for the potential variability in intervention effects between the different HEMS bases in the region. Subgroup ITS analyses regarding response time changes for each single base were however considered to be beyond the scope of this study. Another major weakness of the study is the unbalanced study periods, with a two-year preintervention period and one-year follow-up postintervention. Power in ITS analysis has been shown to increase with an increased number of time points included, increased effect size (the magnitude of impact), slope change as the chosen impact model and balanced study periods [41, 42]. The main reason for this chosen period imbalance in our study is the COVID-19 pandemic, which hit Norway in March 2020. The pandemic has presumably affected HEMS response times in various manners due to necessary clarifications regarding the patient`s COVID-19 status during the dispatch process and extra operational precautions before patient arrival. As such, the pandemic represented an “intervention” itself and could blur the interpretation of the HEMS coordinator time series. The effect of the COVID-19 pandemic on prehospital emergency medical services, including response times, could, however, be a relevant topic for future studies in this field of research.

## Conclusion

The introduction of a regional HEMS coordinator in Mid-Norway did not cause prolonged response times in acute, primary HEMS missions in the region during the first year after the intervention. For one HEMS, the unit`s service area increased slightly, while for two HEMS bases, the service areas were reduced. The proportions of in-flight scrambles did not change. A higher proportion of patients with severe illness or injury was found in the postintervention group. Follow-up analyses are needed to examine if these findings are persistent. A combination of an interrupted time series analysis and the convex-hull method is suitable for evaluating system interventions in the prehospital setting, and could act as a methodological framework for future studies in the field.

## Data Availability

The datasets analysed during the current study are not publicly available due to personally identifiable information, but are available from the corresponding author upon reasonable request.

## Abbreviations

HEMS: Helicopter emergency medical services
GPS: Global positioning system
ITS: Interrupted time series
NACA: National advisory committee for aeronautics
EMCC: Emergency medical communication centre
EMS: Emergency medical services
SAR: Search and rescue
GP: General practitioner
AMIS: Acute medicine information system
ARIMA: Autoregressive integrated moving average
AIC: Akaike information criterion
SD: Standard deviation
IQR: Interquartile range
HEMIT: Central Norway regional health authority information technology
ECG: Electrocardiogram
PCI: Percutaneous coronary intervention
COVID: Coronavirus disease
